# A general modeling framework for quantitative tracking, accurate prediction of ICU, and assessing vaccination for COVID-19 in Chile

**DOI:** 10.3389/fpubh.2023.1111641

**Published:** 2023-03-31

**Authors:** Patricio Cumsille, Oscar Rojas-Díaz, Carlos Conca

**Affiliations:** ^1^Department of Basic Sciences, Faculty of Sciences, University of Bío-Bío, Chillán, Chile; ^2^Centre for Biotechnology and Bioengineering, University of Chile, Santiago, Chile; ^3^Department of Mathematics and Computers Science, Faculty of Science, University of Santiago of Chile, Santiago, Chile; ^4^Department of Mathematical Engineering and Center for Mathematical Modeling, University of Chile (UMI CNRS 2807), Santiago, Chile

**Keywords:** COVID-19, predictive modeling, epidemiological modeling, time delays, vaccination, practical identifiability, parameter optimization, parametric bootstrap

## Abstract

**Background:**

One of the main lessons of the COVID-19 pandemic is that we must prepare to face another pandemic like it. Consequently, this article aims to develop a general framework consisting of epidemiological modeling and a practical identifiability approach to assess combined vaccination and non-pharmaceutical intervention (NPI) strategies for the dynamics of any transmissible disease.

**Materials and methods:**

Epidemiological modeling of the present work relies on delay differential equations describing time variation and transitions between suitable compartments. The practical identifiability approach relies on parameter optimization, a parametric bootstrap technique, and data processing. We implemented a careful parameter optimization algorithm by searching for suitable initialization according to each processed dataset. In addition, we implemented a parametric bootstrap technique to accurately predict the ICU curve trend in the medium term and assess vaccination.

**Results:**

We show the framework's calibration capabilities for several processed COVID-19 datasets of different regions of Chile. We found a unique range of parameters that works well for every dataset and provides overall numerical stability and convergence for parameter optimization. Consequently, the framework produces outstanding results concerning quantitative tracking of COVID-19 dynamics. In addition, it allows us to accurately predict the ICU curve trend in the medium term and assess vaccination. Finally, it is reproducible since we provide open-source codes that consider parameter initialization standardized for every dataset.

**Conclusion:**

This work attempts to implement a holistic and general modeling framework for quantitative tracking of the dynamics of any transmissible disease, focusing on accurately predicting the ICU curve trend in the medium term and assessing vaccination. The scientific community could adapt it to evaluate the impact of combined vaccination and NPIs strategies for COVID-19 or any transmissible disease in any country and help visualize the potential effects of implemented plans by policymakers. In future work, we want to improve the computational cost of the parametric bootstrap technique or use another more efficient technique. The aim would be to reconstruct epidemiological curves to predict the combined NPIs and vaccination policies' impact on the ICU curve trend in real-time, providing scientific evidence to help anticipate policymakers' decisions.

## 1. Introduction

The COVID-19 pandemic has induced a significant research effort for tracking, prediction, and control. In Chile, which is no stranger to the above, health authorities initiated vaccination in the summer of 2021, gradually reducing overall ICU patients and death by COVID-19 while suspending non-pharmaceutical interventions (NPIs) such as lockdowns (partial or total). One of the main lessons is that we must be prepared to face another pandemic like it. Consequently, this article aims to develop a general modeling framework consisting of epidemiological modeling generalization and devising a practical identifiability approach to assess combined vaccination and NPIs strategies for the dynamics of any transmissible disease. To validate the framework, we applied it to track COVID-19 dynamics, accurately predict the ICU curve trend in the medium term, and assess vaccination in Chile.

The literature on COVID-19 modeling is vast. A search in the Web of Science (WOS) with the terms “COVID-19”, “modeling”, and “time delays”, refined by WOS categories related to STEM disciplines, resulted in 95 articles (in January 2023). Therefore, we only review some of those that we believe are important for their applications. For example, Al-Tuwairqi and Al-Harbi (1) proposed a model to investigate the effects of time delay in vaccine production on COVID-19 spread. In addition, Zhenzhen et al. (2) studied a model with “long memory” to describe the multi-wave peaks of the COVID-19 dynamics, where “long memory” allows for predicting this last using non-local terms, which means that one can include an arbitrary long history of the disease. Indeed, for a particular non-local term, the authors obtained a model with time delays. Furthermore, the authors modeled vaccination as an impulsive term that translates into decreased susceptibility.

Moreover, Ghosh et al. (3) derived a model with time delay, where the last is the disease duration, i.e., the average time in which infected individuals recover or die. Zhai et al. (4) investigated a SEIR-type model with time delay and vaccination control. The first parameter is similar to that introduced in our previous work (5) but is considered in the exposed population equation. They simulated vaccination as a control that decreases susceptibility, similar to the generalization we propose in the present work. Finally, our current work relies on generalizing the model developed by (5), which has common elements with some cited works here. Indeed, in (5), we introduced the same time delay that models the average time to recover or die, as in (3). At the same time, we could interpret our previous model (5) as one incorporating a long memory effect in the sense that it allows the reproduction of multi-wave peaks depending on the parameter values, as we showed.

The general goal is to implement a hybrid approach (6), in this case, a holistic combination of mathematical modeling with a practical identifiability approach to reconstruct and predict epidemiological curves based on careful optimization, synthetic data, automatic data scanning, and calibration. Precisely, the scientific novelty of the article relies on developing a general modeling framework that could contribute to anticipating epidemiological scenarios, evaluating the impact of combined vaccination and NPI strategies for any transmissible disease, and helping to visualize the potential effects of implemented plans by policymakers.

To achieve the general goal, we rely on our previous work that forecasted COVID-19's second wave in May 2021 in Chile, calibrating data between March and September 2020 (before vaccination began) through suitable epidemiological modeling (5). Our specific goals are:

1. To generalize our previously developed epidemiological modeling to describe vaccination and assess combined vaccination and NPI strategies for the dynamics of any transmissible disease and, as a study case, of COVID-19.
2. To improve the practical identifiability approach to calibrate the generalized epidemiological modeling with different datasets representing different regions of Chile and accurately forecast the ICU curve trend in the medium term in any stage of the COVID-19 pandemic.
3. To provide open-source codes that implement our general epidemiological modeling framework with standardized parameter initialization for every dataset for reproducibility.

To implement the general modeling framework, we processed and used the official COVID-19 datasets provided by the Chilean government. The framework consists of two main parts: epidemiological modeling generalization and devising a practical identifiability approach. The first consists of a non-linear delay differential equations (DDE) system describing time variation and transitions between the compartments of susceptible, infected, recovered and the sum of ICU plus dead. The second relies on parameter optimization (5, 7, 8), a parametric bootstrap technique (9), and data processing. A novelty of this work is the implementation of a careful parameter optimization algorithm by searching for suitable initializations according to each processed dataset. In addition, we implemented a parametric bootstrap technique to accurately predict the ICU curve trend in the medium term and assess vaccination.

We have organized the article as follows: we describe the general modeling framework in Section 2. More precisely, based on our previous work (5), we describe the epidemiological modeling generalization in Section 2.1. Then, in Section 2.2, we detail the careful parameter optimization algorithm implementation, the parametric bootstrap technique, and data processing, among other methodology pieces. Then, we provide the modeling results to validate the framework with several datasets representing COVID-19 dynamics in different regions of Chile in Section 3. Finally, in Section 4, we discuss the results and give some conclusions in Section 5.

## 2. General modeling framework' description

In this section, we describe the general modeling framework. To do so, we have split it into two main subsections that generalize previously developed epidemiological modeling and devise the practical identifiability approach.

### 2.1. Epidemiological modeling generalization

In the present section, we describe the epidemiological modeling introduced by (5), then present and provide a complete description of each part of the modeling generalization.

#### 2.1.1. Previous work

To derive the epidemiological modeling generalization, we rely on the *generalized SIR model with constant time delays* or *generalized SIR model* previously devised by (5), which describes the NPIs' effect through variations in the rate of disease transmission. We remark here that the NPIs' impact consists of social distancing and that the generalized SIR model does not describe vaccination. To be precise, the model corresponds to the following non-linear DDE system:


(1a) $$\begin{array}{lll} \frac{dS}{dt}\left(t\right) & = & -\frac{\beta \left(t\right)}{N}S\left(t\right)I\left(t-\tau_{1}\right), \end{array}$$

(1b) $$\begin{array}{lll} \frac{dI}{dt}\left(t\right) & = & \frac{\beta \left(t\right)}{N}S\left(t\right)I\left(t-\tau_{1}\right)-\gamma_{IR}I\left(t-\tau_{2}\right), \end{array}$$

(1c) $$\begin{array}{lll} \frac{dR}{dt}\left(t\right) & = & \gamma_{IR}I\left(t-\tau_{2}\right). \end{array}$$

It is worth noting that the generalized SIR model can generate complex dynamics since, by contrast to the classical SIR model, it can simulate more than one local maximum for the infected. Then, it provides a way to explain several COVID-19 waves, depending on the parameters' values (5). In addition, the generalized SIR model would produce better prediction results than the classical SEIR model since no observation of the exposed population is available since, similarly to the asymptomatic population, those exposed are challenging to observe. Indeed, the Chilean government's COVID-19 database (10), apart from the symptomatic cases, counts the asymptomatic ones, and there is no way to know how many of these become symptomatic (5).

The parameters of the model (1) are as follows: β(*t*) corresponds to the mean rate of disease transmission, γ_*IR*_ is the mean removal rate, τ_1_ is the mean incubation time of disease, and τ_2_ is the mean time from onset to clinical recovery or death caused by disease, or the duration time of disease until recovery or death.

Model (1) follows the susceptible-infectious-removed (SIR) paradigm. Susceptible (*S*) individuals infected by SARS-CoV-2 undergo incubation during a mean time (τ_1_) before becoming infected (*I*). The infected individuals are infected by the disease for a mean time (τ_2_) until being removed (*R*) by clinical recovery or death.

The initial conditions have to satisfy *S*(*t*_0_) + *I*(*t*_0_) + *R*(*t*_0_) = *N*, where *N* is the size of the population under study for a closed system and taking into account that (*S* + *I* + *R*)′(*t*) = 0 for all *t* > 0, for a suitably chosen *t*_0_.

In the case of COVID-19, following the discussion by (5), we assume that the number of infected reported with symptoms confirmed by Reverse Transcriptional Polymerase Chain Reaction (RT-PCR) tests, denoted by *I*_*r*_(*t*), is underestimated since it depends on the availability and application of RT-PCR tests. Consequently, we assume that *I*_*r*_(*t*) is a fraction of the *actual number of infected* *I*(*t*),

(2) $$\begin{array}{l} I_{r}\left(t\right)=f\left(t\right)I\left(t\right), \end{array}$$

where *f*(*t*) is the ratio of positive RT-PCR tests number (confirmed cases) over the actual infected cases for the day *t*, which accounts for the *real positivity rate*. It is worth noting that *f*(*t*) is not the same as the positivity rate of detected cases, but it is related to it, and it involves the asymptomatic infected (5). We modeled *f*(*t*) as an inverted Sigmoid-type function such that if *I*(*t*) is small enough, which occurred during the beginning of the outbreak, then an important fraction of the real infected cases are detected (*I*_*r*_(*t*) ≈ *I*(*t*)). On the contrary, when *I*(*t*) is large enough, which occurred just before the quarantines were imposed, then only a small fraction 0 < *a* < 1 of the real infected cases are detected (*I*_*r*_(*t*) ≈ *aI*(*t*)). Precisely, *f*(*t*) is defined as

(3) $$\begin{array}{l} f\left(t\right)=1+\frac{a-1}{1+e^{-k\left(I\left(t\right)-I_{thr}\right)}} \end{array}$$

whose parameters are *a*, *k* and *I*_*thr*_, where *a* and *k* represent the minimum and the decay rate of *f*(*t*), respectively. On the other hand, to measure how large/small *I*(*t*) is, we introduce a threshold *I*_*thr*_ such that *I*(*t*)≪*I*_*thr*_ implies *f*(*t*) ≈ 1, and *I*(*t*) ≫ *I*_*thr*_ implies *f*(*t*) ≈ *a* ≪ 1.

Model (1), describing time variation and transitions *S*-to-*I*-to-*R* plus Equations (2)–(3), representing the real positivity rate, is quite general since it models the dynamics of any transmissible disease, not considering vaccination. The model parameters, gathered in vector (β(*t*), γ_*IR*_, τ_1_, τ_2_, *a, k, I*_*thr*_), are unknown or inaccessible and have to be identified from time-series observations *I*_*r*_ corresponding to the number of infected reported with symptoms confirmed by RT-PCR tests. In (5), we devised a practical identifiability approach, i.e., a set of techniques to reliably estimate parameters with acceptable accuracy from noisy data (11, 12). In particular, we reproduced epidemiological scenarios considering β(*t*) varying in time to describe NPI strategies ranging from total relaxation to imposing strict social distancing (complete lockdown differed by municipalities) at different periods. As a result, we forecasted the second wave of May 2021 in Chile, calibrating data between March and September 2020 (before vaccination began).

#### 2.1.2. Toward modeling's generalization

To make model (1) more complete and realistic, we developed a generalization to measure the hospital load on the healthcare system through the number of patients hospitalized in the ICU and to assess the vaccination. Equations (2)–(3) that model the real positivity rate remain unchanged. In the following, we provide a modeling description of both aspects.

##### 2.1.2.1. Vaccination description

According to (13), vaccination protects in four ways: against infection, symptoms, severe disease, and reducing onward transmission. However, even considering part of the vaccination's ways of protection, the model can become very complex, as in the work by (13). Moreover, since COVID-19 data has great uncertainty, among other issues discussed in the data processing section, any model will provide results accordingly, no matter how exact its representation of reality is. Consequently, our present work is in the same spirit as our previous work (5) in that keeping a model simple is critical to reasonably carrying out a practical identifiability approach. Even so, it is still a challenge to do it in real-time to anticipate epidemiological scenarios to help predict the hospital load on the healthcare system, as required in the first year of the pandemic (before vaccination). In this regard, we assume vaccination protects against infection by diminishing susceptibility, as assumed, for example, by (2, 4), which translates into adding new terms to Equations (1a) and (1c) for those susceptible and those recovered and several meaningful parameters associated with vaccination.

According to the previous discussion, the new terms that model vaccination account for the transition from susceptible to recovered, its inverse, and their respective times of transition. Transition rates are denoted by γ_*SR*_(*t*) and γ_*RS*_(*t*), while the times model as delays within the new terms added, designated by τ_3_ and τ_4_, respectively. We allow transition rates to vary in time to describe them more realistically since they depend on several factors that may change over time, as explained below (e.g., the immunity of an individual without booster doses decays faster).

Parameter γ_*SR*_(*t*) relates mainly to the vaccination uptake rate, while γ_*RS*_(*t*) relates to the waning of immunity after vaccination. Moreover, parameters τ_3_ and τ_4_ depend on every delivered vaccine's effectiveness and immunity waning. However, the vaccination uptake rate, effectiveness, and immunity waning of vaccines are not well-determined since they depend on several factors such as prior infection, age, sex, T-cell response, and the periodicity of vaccine injections. In addition, only natural infection mounts a significant and lasting immune response (14). Therefore, parameters γ_*SR*_(*t*), γ_*RS*_(*t*), τ_3_, and τ_4_ depend on many factors, which makes it difficult to estimate them and they are strongly available data-dependent, independent of how exact the model's representation of reality is. Thus, they are inaccessible and must be identified from time-series observations of COVID-19, as noted before.

The model equations that consider vaccination are:

(4a) $$\begin{array}{l} \frac{dS}{dt}\left(t\right)=-\frac{\beta \left(t\right)}{N}S\left(t\right)I\left(t-\tau_{1}\right)-\gamma_{SR}\left(t\right)S\left(t-\tau_{3}\right)+\gamma_{RS}\left(t\right)S\left(t-\tau_{4}\right), \end{array}$$

(4b) $$\begin{array}{c} \frac{dR}{dt}\left(t\right)=\gamma_{IR}\left(t\right)I\left(t-\tau_{2}\right)\\ +\gamma_{SR}\left(t\right)S\left(t-\tau_{3}\right)-\gamma_{RS}\left(t\right)S\left(t-\tau_{4}\right)+\gamma_{UR}\left(t\right)U\left(t-\tau_{6}\right). \end{array}$$

The second and third terms of Equations (4a)–(4b) model the transitions from the susceptible to recovered compartment, and conversely, τ_3_ stands for the mean time delay for those susceptible to become immune after vaccination, and γ_*SR*_(*t*) indicates how fast it happens. Similarly, τ_4_ designates the mean time until an individual loses immunity, so τ_4_ is the mean duration of immunity by vaccination, and γ_*RS*_(*t*) measures how fast it happens. Finally, the last term in Equation (4b) pertains to the *U* compartment, which we explain below.

##### 2.1.2.2. *U* compartment description and modeling generalization summary

Finally, as mentioned before, we added the variable *U* to model (1), representing the sum of the patients in the ICU plus those confirmed dead due to COVID-19, the equation of which contains the transitions from *I*-to-*U* and *U*-to-*R*. The variable *R* now describes the recovered, whereas *R* in the model (1) represented the removed, i.e., the sum of those who had recovered plus those who had died.

As before, the mentioned transitions encompass rates and time delays, denoted by γ_*IU*_(*t*), τ_5_, γ_*UR*_(*t*), and τ_6_. Precisely, we model the transition *I*-to-*U* by the rate γ_*IU*_(*t*) and the time τ_5_ that those infected took to be admitted to the ICU. In addition, we represent the transition *U*-to-*R* by the rate γ_*UR*_(*t*) and the time τ_6_ that patients took to recover in the ICU.

We summarize the modeling generalization as the following non-linear DDE system:


(5a) $$\begin{array}{l} \frac{dS}{dt}\left(t\right)=-\frac{\beta \left(t\right)}{N}S\left(t\right)I\left(t-\tau_{1}\right)-\gamma_{SR}\left(t\right)S\left(t-\tau_{3}\right)+\gamma_{RS}\left(t\right)S\left(t-\tau_{4}\right), \end{array}$$

(5b) $$\begin{array}{l} \frac{dI}{dt}\left(t\right)=\frac{\beta \left(t\right)}{N}S\left(t\right)I\left(t-\tau_{1}\right)-\gamma_{IR}\left(t\right)I\left(t-\tau_{2}\right)-\gamma_{IU}\left(t\right)I\left(t-\tau_{5}\right), \end{array}$$

(5c) $$\begin{array}{c} \frac{dR}{dt}\left(t\right)=\gamma_{IR}\left(t\right)I\left(t-\tau_{2}\right)+\gamma_{SR}\left(t\right)S\left(t-\tau_{3}\right)-\gamma_{RS}\left(t\right)S\left(t-\tau_{4}\right)\\ +\gamma_{UR}\left(t\right)U\left(t-\tau_{6}\right), \end{array}$$

(5d) $$\begin{array}{l} \frac{dU}{dt}\left(t\right)=\gamma_{IU}\left(t\right)I\left(t-\tau_{5}\right)-\gamma_{UR}\left(t\right)U\left(t-\tau_{6}\right). \end{array}$$

Again, the previous system is closed since the variables' sum equals *N*, the size of the targeted population. The DDE system (5) and Equations (2)–(3) will be named *general epidemiological modeling*, which is quite broad since it models the dynamics of any transmissible disease under any combination of NPIs and vaccination. To describe simply the in-time-variation of parameters β(*t*), γ_*IU*_(*t*), and γ_*UR*_(*t*), we assumed that these are piecewise linear functions. Then, the functions β(*t*), γ_*IU*_(*t*), and γ_*UR*_(*t*) are represented by the vectors ***β***, ***γ***_***IU***_ and ***γ***_***UR***_ in ${\mathbb{R}}^{n_{\beta }+1}$ that represent *n*_***β***_ straight lines approximating the respective functions. We gave the same description for the in-time-variation of parameters γ_*SR*_(*t*), γ_*RS*_(*t*), and γ_*IR*_(*t*), i.e., they are represented by the vectors ***γ***_***SR***_, ***γ***_***RS***_, and ***γ***_***IR***_ in ${\mathbb{R}}^{n_{\gamma }+1}$.

Therefore, general epidemiological modeling depends on *p*: = 3(*n*_***β***_ + *n*_***γ***_) + 15 parameters gathered in the vector ***θ*** ∈ ℝ^*p*^ defined by:


(6a) $$\begin{array}{l} \theta =\left(\theta_{1},\;\theta_{2}\right)\in {\mathbb{R}}^{p}\;, \end{array}$$

(6b) $$\begin{array}{l} \theta_{1}={\left(\gamma_{IR},\;\gamma_{SR},\;\gamma_{RS},\;\gamma_{IU},\;\beta ,\;\gamma_{UR},\;\tau \right)}^{t}\in {\mathbb{R}}^{3\left(n_{\gamma }+n_{\beta }\right)+12},\; \end{array}$$

(6c) $$\begin{array}{l} \gamma_{IR},\;\gamma_{SR},\;\gamma_{RS}\in {\mathbb{R}}^{n_{\gamma }+1}, \end{array}$$

(6d) $$\begin{array}{l} \beta ,\;\gamma_{IU},\;\gamma_{UR}\in {\mathbb{R}}^{n_{\beta }+1}, \end{array}$$

(6e) $$\begin{array}{l} \tau ={\left(\tau_{1},\;\tau_{2},\;\tau_{3},\;\tau_{4},\;\tau_{5},\;\tau_{6}\right)}^{t}\in {\mathbb{R}}^{6}, \end{array}$$

(6f) $$\begin{array}{l} \theta_{2}={\left(a,\;k,\;I_{thr}\right)}^{t}\in {\mathbb{R}}^{3}, \end{array}$$

where *n*_***γ***_, *n*_***β***_ is the number of time intervals to reconstruct γ_*SR*_(*t*), γ_*RS*_(*t*), and γ_*IR*_(*t*), and β(*t*), γ_*IU*_(*t*), γ_*UR*_(*t*) piecewise linearly with equally spaced intervals, respectively. For instance, using *n*_***β***_ = 9, *n*_***γ***_ = 9, one has *p* = 3(*n*_***β***_ + *n*_***γ***_) + 15 = 69 parameters to estimate, i.e., ***θ*** ∈ ℝ^69^.

Table 1 summarizes the parameters of general epidemiological modeling given by Equations (5), (2), and (3).

**Table 1:** Parameters of general epidemiological modeling.

**Symbol**	**Description**	**Unit**
*τ*_1_	Mean incubation time	days
*τ*_2_	Mean time to recover for mild cases	days
*τ*_3_	Mean time from susceptible to recovery (by vaccination immunity)	days
*τ*_4_	Mean duration of immunity (by vaccination)	days
*τ*_5_	Mean time from infected to ICU	days
*τ*_6_	Mean time from ICU to recover	days
***γ***_***IR***_	Mean recovery rate for mild cases	days^−1^
***β***	Mean transmission rate	days^−1^
***γ***_***IU***_	Mean transition rate from infected to ICU	days^−1^
***γ***_***UR***_	Mean recovery rate for ICU patients	days^−1^
***γ***_***SR***_	Mean transition rate from susceptible to recovered	days^−1^
***γ***_***RS***_	Mean transition rate from recovered to susceptible	days^−1^
*a*	Minimum of the real positivity rate	–
*k*	Decay rate of the real positivity rate	inhabitants^−1^
*I*_*thr*_	Infection threshold of the real positivity rate	inhabitants

### 2.2. A practical identifiability approach

We devised a practical identifiability approach that relies on parameter optimization, a parametric bootstrap technique, and data processing, for which computer implementation includes open-source data and code repository through GitHub (15). The approach relies on solving a parameter estimation problem through a careful optimization algorithm, numerical resolution of modeling equations, a parametric bootstrap technique, and data processing. For solving the modeling equations, we required the provision of reasonable bounds for the parameters, which is critical for achieving a stable numerical method. In addition, the parameter range is meaningful, at least regarding the time delays that describe relevant parameters from the epidemiology viewpoint.

Next, we describe each piece of the practical identifiability approach.

#### 2.2.1. Parameter estimation problem description

To reproduce and predict COVID-19 dynamics in Chile, one has to solve the *parameter estimation problem:* given a dataset of the time-series observations of COVID-19 dynamics, identify the parameter vector ***θ*** such that general epidemiological modeling fits them in the least-squares sense. The time-series observations we used are


$$\left\{\left[{\left(I_{r}\right)}_{j},\;{\left(U_{r}\right)}_{j}\right]\;:\;j=1,\cdots \;,n\right\}.$$

*I*_*r*_ corresponds to the number of infected reported with symptoms confirmed by RT-PCR tests [see Equation (2) and its respective explanation], *U*_*r*_ corresponds to the observations of variable *U*, i.e., the sum of the patients in the ICU and confirmed deaths due to COVID-19, and *n* is the number of data.

More precisely, we have to find the vector ***θ*** ∈ ℝ^*p*^, defined by Equation (6), that minimizes the sum of squares:


(7) $$\begin{array}{l} \text{SS}\left(\theta \right):=||{\left(Res_{I},\;Res_{U}\right)}^{t}|{|}^{2}=\overset{n}{\underset{j=1}{\sum }}\left[{\left(Res_{I}\right)}_{j}^{2}+{\left(Res_{U}\right)}_{j}^{2}\right] \end{array}$$

where *Res*_*I*_ and $Res_{U}\in {\mathbb{R}}^{n}$ stand for the relative residuals of variable *I* and *U*, respectively, defined by


(8a) $$\begin{array}{lll} {\left(Res_{I}\right)}_{j} & :=\frac{\left[{\left(I_{r}\right)}_{j}-f\left(t_{j},\theta_{2}\right)I\left(t_{j},\theta_{1}\right)\right]}{f\left(t_{j},\theta_{2}\right)I\left(t_{j},\theta_{1}\right)} & j=1,\cdots \;,n \end{array}$$

(8b) $$\begin{array}{lll} {\left(Res_{U}\right)}_{j} & :=\frac{\left[{\left(U_{r}\right)}_{j}-U\left(t_{j},\theta_{1}\right)\right]}{U\left(t_{j},\theta_{1}\right)} & j=1,\cdots \;,n. \end{array}$$

The objective function defined in (7) corresponds to the sum of squares of the residuals relative to the model observations, *f*(*t*_*j*_, ***θ***_2_)*I*(*t*_*j*_, ***θ***_**1**_) and *U*(*t*_*j*_, ***θ***_**1**_). The choice of the relative residuals obeys to take into account the unequal quality of the observations (16). In this case, the patients in the ICU plus confirmed deaths due to COVID-19 (variable *U*) is better observed than those infected (variable *I*).

The components of *Res*_*I*_, defined in (8a), correspond to the differences between the time-series observations (*I*_*r*_)_*j*_, and the model observations *f*(*t*_*j*_, ***θ***_**2**_)*I*(*t*_*j*_, ***θ***_**1**_) (see Equation 2). The components of *Res*_*U*_, defined in (8b), are the differences between the time-series observations (*U*_*r*_)_*j*_, and the model observations *U*(*t*_*j*_, ***θ***_**1**_). Both variables, *I* and *U*, correspond to the solution of the general epidemiological model (5), (2), and (3) calculated at (*t*_*j*_, ***θ***) for *j* = 1, ⋯ , *n*, for a given parameter vector $\theta =\left(\theta_{1},\;\theta_{2}\right)\in {\mathbb{R}}^{p}$ defined by (6).

#### 2.2.2. A careful optimization algorithm

To calibrate general epidemiological modeling, we implemented a careful optimization algorithm that combines data processing algorithms (cleaning, smoothing, and curve interpolation), initial parameter estimation generation, and a non-linear least-squares optimization method to minimize *SS*(***θ***) [Equation (7)] for each processed dataset.

The argument minimum of the sum of squares designs as $\hat{\theta }$ for every dataset. The vector $\hat{\theta }$ is called the *non-linear least-squares estimator*, abbreviated as *non-linear LSE*.

Next, we provide details on the implementation.

##### 2.2.2.1. Implementation

We employed the *Trust-Region Interior Reflective* (TIR) method implemented in Matlab R2022b as the subroutine *lsqnonlin*, specially adapted for solving non-linear least-squares minimization problems. The convergence of the TIR method depends strongly on initial parameter estimation, which has to be relatively close to the optimal solution (7, 8). We efficiently minimized the objective function by implementing a *percentage decrease technique* from the parameters' ranges to calculate suitable initial parameter vectors for every dataset in a standardized manner. According to our previous experience (5), the initial parameters that mainly influence the fitting results are $\beta ,\;\gamma_{IU},\;\gamma_{UR}\in {\mathbb{R}}^{n_{\beta }+1}$, which describe the mean rate of disease transmission and transitions from *I*-to-*U* and *U*-to-*R*. To avoid overfitting, we selected *n*_***β***_ equispaced intervals to accurately fit and predict after the final calibration time for every dataset.

Next, we explain the numerical resolution of general epidemiological modeling within a range for meaningful parameters and the percentage decrease technique, which are critical for implementing our careful optimization algorithm.

##### 2.2.2.2. Numerical resolution of general epidemiological modeling

To evaluate the objective function *SS*(***θ***), we numerically solved the model (5) at *t*_*j*_, *j* = 1, ⋯ , *n*, for different parameter vectors ***θ*** chosen *ad-hoc* for each dataset. We carried out the numerical resolution by a Runge-Kutta type formula (17): the subroutine *dde23* implemented in Matlab, designed for solving non-linear DDE systems. In addition, we reconstructed the function of history (required for solving DDE instead of the initial condition for classical ordinary differential equations systems) for the model (5) by interpolating the data *I*_*r*_(*t*), *U*_*r*_(*t*), and recovered for every studied dataset. We used a shape-preserving piecewise cubic interpolation as devised by the *interp1* Matlab subroutine with the option *pchip*.

##### 2.2.2.3. Meaningful parameter bounds

We computed the model parameters ***θ*** given in (6) using meaningful bounds from an epidemiological viewpoint for the time delays: 1 ≤ τ_1_ ≤ 14, 1 ≤ τ_2_ ≤ 21, 14 ≤ τ_5_ ≤ 56, and 21 ≤ τ_6_ ≤ 42 days. We imposed these bounds because the incubation period (τ_1_) ranges from 1 to 14 days (mean of 5–6 days), the median time from onset to clinical recovery for mild COVID-19 cases is approximately 2 weeks (τ_2_), and is 3–6 weeks for patients with severe or critical symptoms (τ_6_). In addition, among patients who died, the time from symptom onset to outcome ranged from 2 to 8 weeks (τ_5_) (18). In addition, we assume that vaccination immunity duration, τ_4_, ranges from 1 to 240 days since immunity declines only at 6–8 months after natural infection (19). In contrast, no range is well-determined for the transition time from susceptible to recovered, τ_3_, however, one may expect that it is relatively small, so we assume 1 ≤ τ_3_ ≤ 14.

For the real positivity fraction, *f*(*t*), one has that

$$\begin{array}{r} 0<a<1,\;\min\left\{{\left(I_{r}\right)}_{j}\;:\;j=1,\cdots \;,n\right\}\leq I_{thr}\leq \max\{{\left(I_{r}\right)}_{j}\;:\;\\ j=1,\cdots \;,n\}. \end{array}$$

Finally, all the rest of the parameters (transition and transmission rates) have to be within ranges to achieve stability of the careful optimization algorithm implementation, mainly related to general epidemiological modeling numerical resolution, as we explain next.

##### 2.2.2.4. Percentage decrease technique for numerical stability

Convergence of the optimization algorithm, lsqnonlin, depends directly on that of the dde23 solver, both implemented in Matlab. Through our experiments, we verified that the model's numerical solution calculated by dde23 strongly depends on the derivatives of the first points evaluated, and its convergence relies on the closeness of the initial curves used to build the history function. To overcome this stability problem without intervening or designing new Matlab numerical libraries, we developed a simple but effective technique, called the *percentage decrease technique*, to produce initial curves contained in the feasible space of the official data curves.

The percentage decrease technique consists of multiplying the upper bounds of the parameter vector ***θ*** (see Equation 6) by a fraction ω ∈ [1*e* − 4, 1*e* − 2], excluding time delays ***τ*** (see Equation 6e). Concretely, we chose the initial parameter vector defined by $\theta^{\left(0\right)}:=\omega \cdot {\text{UB}}_{\theta }$, where UB_***θ***_ stands for the upper bound of ***θ***, described in Section 2.2.2.3.

We calibrated ω for every studied dataset representing a characteristic epidemiological curve, with the magnitude or period of peaks' duration differentiated, which is mainly related to the density and mobility of the population. We chose a value of ω inversely proportional to the population size within the interval [1*e* − 4, 1*e* − 2]. Therefore, one should use a small value, e.g., ω = 1*e* − 3 or 0.1% of UB_***θ***_ for large cities (millions of inhabitants), and an even smaller value, e.g., ω = 1*e* − 4 or 0.01% of UB_***θ***_ for towns with fewer inhabitants (less than one million). Generally, ω = 1*e* − 3 works well for all cases obtaining a similar minimum error. Still, we calibrated a suitable value of ω for every dataset to speed up the execution time of the optimization subroutine lsqnonlin.

#### 2.2.3. A parametric bootstrap technique

Once we calibrated a dataset, we applied a parametric bootstrap technique (PBT) to quantify the parameters' uncertainty and construct confidence intervals to achieve reliable and accurate forecasting performance, obtained by propagating the uncertainty (9). The PBT generates synthetic datasets repeatedly sampled from the least-squares curves:

(9) $$\begin{array}{l} F\left(t_{j},\hat{\theta }\right):=\left[f\left(t_{j},{\hat{\theta }}_{2}\right)I\left(t_{j},{\hat{\theta }}_{1}\right),\;U\left(t_{j},{\hat{\theta }}_{1}\right)\right]. \end{array}$$

However, the PBT requires intensive computational resources and time since it generates simulated data from $F\left(t_{j},\hat{\theta }\right)$ and calculates least-squares parameter estimates for each generated synthetic dataset. Therefore, we applied this technique to show the predictive power of general epidemiological modeling and assess vaccination only for one dataset, corresponding to days 280–530 that encompass the Metropolitan region's second wave, and considering the last 12 weeks to test the forecasting performance of the PBT.

In the PBT, one assumes that synthetic data follows a given probability distribution with an expected value equal to the least-squares curves $F\left(t_{j},\hat{\theta }\right)$. To be precise, we implemented the following algorithm:

1. We calculated the parameter estimates $\hat{\theta }$ through least-squares fitting the model to the time-series data to obtain the best-fit model given by $F\left(t_{j},\hat{\theta }\right)$ (see Equation 9).
2. Using the least-squares fitted model $F\left(t_{j},\hat{\theta }\right)$, we generated *M* replicated *synthetic datasets* denoted by $F_{1}^{SD}\left(t_{j},\hat{\theta }\right),\;F_{2}^{SD}\left(t_{j},\hat{\theta }\right),\;\cdots \;,\;F_{M}^{SD}\left(t_{j},\hat{\theta }\right)$. We generated synthetic datasets as random vectors with a mean equal to $F\left(t_{j},\hat{\theta }\right)$:
  (10) $$\begin{array}{l} F_{k}^{SD}\left(t_{j},\hat{\theta }\right)\sim \text{Dist}\left[F\left(t_{j},\hat{\theta }\right)\right] \end{array}$$
  where **Dist** is a given probability distribution of mean equal to $F\left(t_{j},\hat{\theta }\right)$, and variance proportional to the mean magnitude or the covariance matrix of it. For instance, we used the normal, Poisson, and negative binomial distributions. The last is adequate to model data over-dispersion while controlling its magnitude (9).
3. We re-calculated the least-squares parameter estimates fitting the model to each of the *M*-simulated datasets realizations. Each parameter vector is denoted by ${\hat{\theta }}_{\ell }$ for ℓ = 1, 2, ⋯ , *M*.
4. Using the set of re-estimated parameters ${\hat{\theta }}_{\ell }$, ℓ = 1, 2, ⋯ , *M*, we calculated a confidence interval at the level of 95%. The uncertainty around the least-squares model fit is given by $F\left(t_{j},{\hat{\theta }}_{1}\right),\;F\left(t_{j},{\hat{\theta }}_{2}\right),\cdots \;,\;F\left(t_{j},{\hat{\theta }}_{M}\right)$.

Typical values for the number of bootstrap samples *M* range from 50 to 200 for a proper standard error estimation; see p. 13–14 in (20). Indeed, by choosing *M* = 100, the standard error estimate provides reliable results for parameter estimation, as shown in Section 4. However, beyond choosing a good number of bootstrap samples *M*, careful implementation of the PBT is critical for obtaining ${\hat{\theta }}_{\ell }$ corresponding to $F\left(t,{\hat{\theta }}_{\ell }\right)$ so that the curves that are reproduced and predicted remain positive for all *t* within the time interval of calibration and prediction.

The calculated confidence interval is our prediction interval, denoted by PI, and defined by:


(11a) $$\begin{array}{r} \text{PI}:=\left[{\text{LB}}_{\hat{\theta }},\;{\text{UB}}_{\hat{\theta }}\right]:=\left[\bar{\hat{\theta }}\pm t_{0.975,M-1}\;\text{SE}\right]\\ =\bar{\hat{\theta }}\left[1\pm t_{0.975,M-1}\;\text{NSE}\right], \end{array}$$

(11b) $$\begin{array}{l} \text{NSE}:=\frac{\text{SE}}{\bar{\hat{\theta }}}=\frac{1}{\sqrt{M}}\frac{s_{\hat{\theta }}}{\bar{\hat{\theta }}}, \end{array}$$

where $\bar{\hat{\theta }}\in {\mathbb{R}}^{p}$ is the mean of the *M* bootstrap parameters ${\hat{\theta }}_{\ell }\in {\mathbb{R}}^{p}$, *t*_0.975, *M*−1_ is the 0.975 percentile of the *t*-student distribution with *M* − 1 degrees of freedom, $s_{\hat{\theta }}\in {\mathbb{R}}^{p}$ is the standard deviation of the *M* bootstrap parameters, and **NSE** ∈ ℝ^*p*^ is the normalized standard error (5) (the definitions of $s_{\hat{\theta }}$ and **NSE** are understood component-by-component).

The resulting uncertainty around the least-squares model fit, $F\left(t,\hat{\theta }\right)$, is quantified by the 95% confidence bounds [${\text{LB}}_{\hat{\theta }}$ and ${\text{UB}}_{\hat{\theta }}$, defined by (11)] (9). Since we are interested in computing a more accurate prediction for the ICU curve trend in the medium term, we define an error criterion that includes performance for fit and forecasting. More precisely, we define

(12) $$\begin{array}{r} E\left({\hat{\theta }}_{\ell }\right):=0.6{\text{FP}}_{U}\left({\hat{\theta }}_{\ell }\right)+0.2{\text{FP}}_{I}\left({\hat{\theta }}_{\ell }\right)+0.1[{\text{RMSE}}_{U}\left({\hat{\theta }}_{\ell }\right)\\ +{\text{RMSE}}_{I}\left({\hat{\theta }}_{\ell }\right)],\;\ell =1,\cdots \;,M. \end{array}$$

In Equation (12), the root mean squared error (RMSE) to measure the fit performance is defined by (16):

(13) $$\begin{array}{l} {\text{RMSE}}_{I}={\left[\frac{1}{n-p}\overset{n}{\underset{j=1}{\sum }}{\left(Res_{I}\right)}_{j}^{2}\right]}^{1/2},\\ \;{\text{RMSE}}_{U}={\left[\frac{1}{n-p}\overset{n}{\underset{j=1}{\sum }}{\left(Res_{U}\right)}_{j}^{2}\right]}^{1/2}. \end{array}$$

We calculated the RMSE over the calibration period (*n* and *p* are the calibrated dataset size and the number of model parameters, respectively). A similar criterion was employed for the forecasting performance (FP), but the sum over the prediction period was computed and normalized by the number of predicted data points.

In Equation (12), we gave more weight (60%) to FP_*U*_ since variable *U* is better observed than variable *I*, followed by FP_*I*_ (20%) and the RMSE for both variables (10% each). Furthermore, it was more important to make more effort to follow up with the sum of the ICU patients and those who had died than those who were infected. Thus, we first focused on having better forecasting performance for ICU patients plus those who died and then on the number of infected persons reported. We gave less importance to the fitting performance, so the model did not overfit to actual data, which have much uncertainty, among other problems discussed in Section 3.1.

From the error criterion given in (12), we define the *best parameter vector*, denoted by ${\hat{\theta }}_{\bar{k}}$, among the vectors ${\hat{\theta }}_{\ell }$ for ℓ = 1, ⋯ , *M* that minimizes $E\left({\hat{\theta }}_{\ell }\right)$, i.e.,

(14) $$\begin{array}{l} E\left({\hat{\theta }}_{\bar{k}}\right):=\underset{1\leq \ell \leq M}{\min}E\left({\hat{\theta }}_{\ell }\right) \end{array}$$

The best parameter vector defined in this way realizes the minimum error of the PBT we implemented and induces the *best model curves*, which privilege the forecasting performance. So, the best model curves are those evaluated at the best parameter vector, $F\left(t,{\hat{\theta }}_{\bar{k}}\right)$. In addition, the prediction interval PI produces an envelope of model curves $F\left(t,{\hat{\theta }}_{\ell }\right)$ for ℓ = 1, ⋯ , *M*, quantifying uncertainty around the best model curves $F\left(t,{\hat{\theta }}_{\bar{k}}\right)$.

Finally, to assess the impact of vaccination, we compared different immunity durations, the parameter τ_4_, from 60 to 240 days, spaced every 30 days. For that, we calculated a weighted mean between the least PBT error $E\left({\hat{\theta }}_{\bar{k}}\right)$ given in (14) with the mean of the **NSE** given in (11b), denoted by $\bar{\text{NSE}}$. We evaluated the best τ_4_ as the value minimizing the weighted error, WE, defined by

(15) $$\begin{array}{l} \text{WE}:=\frac{2}{3}E\left({\hat{\theta }}_{\bar{k}}\right)+\frac{1}{3}\bar{\text{NSE}}. \end{array}$$

We gave more weight (66.67%) to the minimum PBT error and the least weight (only 33.33%) to the mean of the NSE to privilege the FP in (14) over the uncertainty represented by the NSE in (11b), which was within reasonable bounds.

#### 2.2.4. Data processing and actual data limitations

The datasets' sources correspond to the Chilean government's COVID-19 database at the regional level, which we used to track and predict COVID-19 dynamics (10). We used datasets representing different regions of the north, south, and center, including the Metropolitan Region (MR), the major city of which is the country's capital, Santiago de Chile. They correspond to reported infected persons with symptoms confirmed by RT-PCR tests (*I*_*r*_), recovered cases (*R*), patients in the ICU, those confirmed dead due to COVID-19 (*D*), and the size of targeted populations (*N*). We applied mobile averages with different window sizes to deal with data that were not always reported daily and to smooth out the data.

##### 2.2.4.1. Available actual data limitations

**Figure 1:**
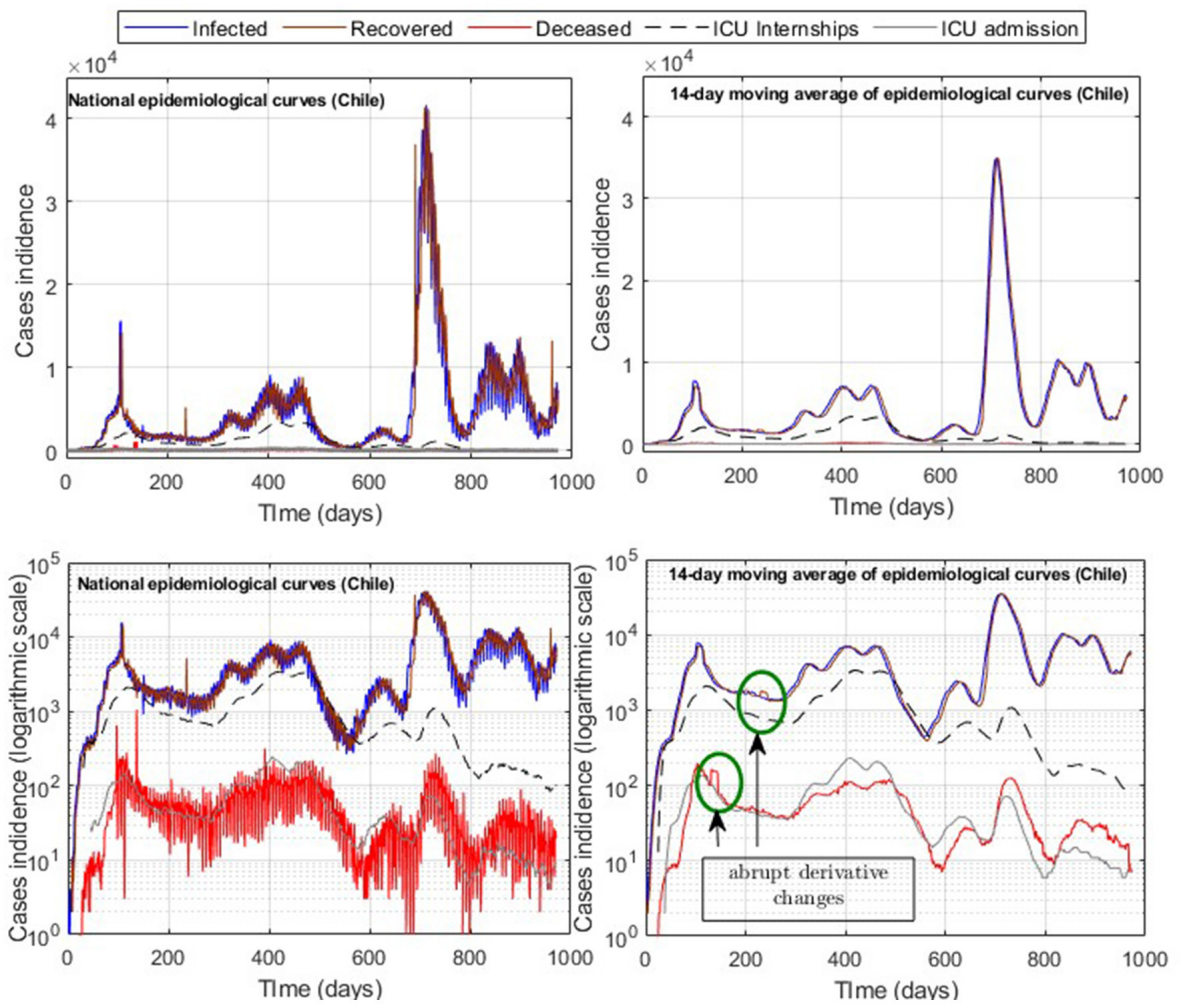
Official COVID-19 incidence curves (Chile); raw data and processing with moving averages.

A common problem in processing and modeling epidemiological curve data is the time lag of the information reported, which was not daily but accumulated every 3–5 days in the Chilean case as observed in Figure 1, which depicts various sub-peaks in the official Chilean data curves, specifically in the data of those infected (*I*), recovered (*R*), and deceased (*D*). In addition, the relevant information is only detailed at the national level, such as daily admissions to the ICU and COVID-19 reinfections according to the vaccination scheme, which was accumulated weekly, and provisional since it was being validated [(10), product 90]. Therefore, using data in constant validation does not ensure a precise replication of the results, unlike *I*, *R*, and *ICU*, which have been maintained without significant variation over time. In addition, in the case of deaths in Chile at the beginning of 2022, the probable cases (without a prior confirmation test for COVID-19) were added to the official data (more than 10,000 cases) for the daily death count. Since the daily distribution of such cases is unknown, in our study, we only considered confirmed death due to COVID-19 reported daily.

**Figure 2:**
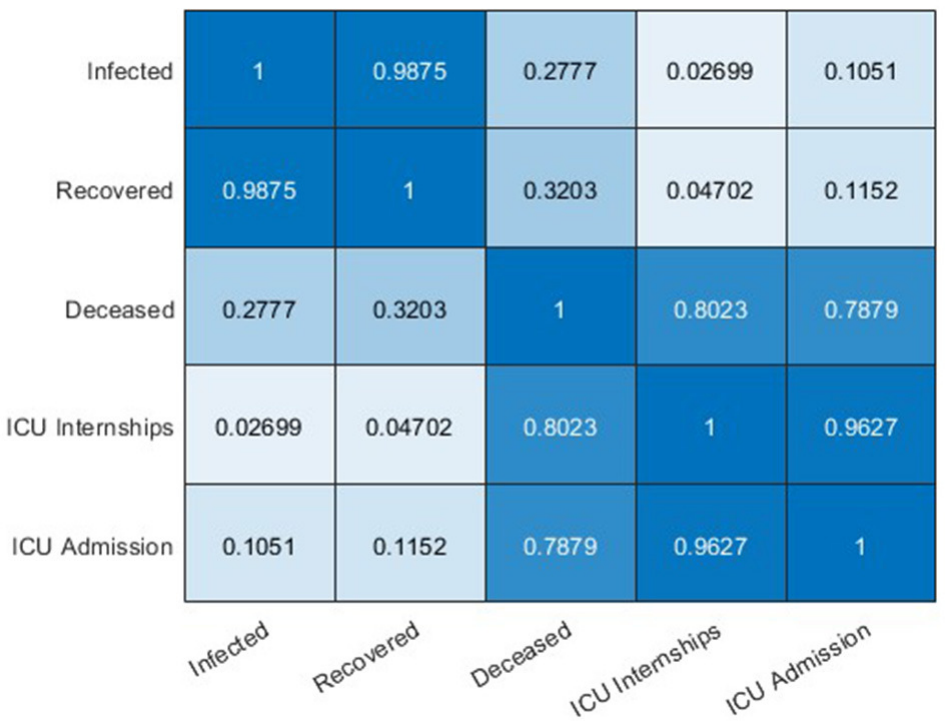
Correlations of incidence curves (Chile).

Although different epidemiological datasets are available in the official Chilean repository (10), most are designed for statistical studies rather than for modeling studies. The most consistent data (without repetition of cases) is deceased persons, which has a high correlation (Pearson coefficient of 0.8) with hospitalized ICU patients (Figure 2). Therefore, in addition to being relevant data to determine the hospital load, hospitalized ICU patients are of high value in terms of the quality of the available data, from which one can conduct modeling studies at the regional level for the Chilean case. Furthermore, the interest in approximating regional curves and not only the national ones is based on the fact that it allows us to validate our general modeling framework with several datasets and analyze the epidemic in different geographical areas. The previous consideration is relevant to Chile, the longest country in the world, with different climates from north to south, and therefore a good case study for the present work. Finally, we applied our general modeling framework to study the COVID-19 data available (*I*, *R*, *ICU*, and *D*) and, in the future, we will extend and adapt to any transmissible disease in any country. It is feasible given that in most of the world, it is more viable to track critical cases (those hospitalized in ICUs), deaths, and, to a lesser extent, those who are infected/recovered (the actual total is never reached).

##### 2.2.4.2. Hardware, software, and parallel computing

We used a data science workstation for the careful optimization algorithm implementation with the following features: Intel Core i9 7900x, 10 Cores/20 threads, 128 Gb memory, NVIDIA Titan RTX 24 Gb, and two mobile laptops with Intel Core i7s, 4 Cores/8 threads with 16 Gb memory. We implemented all our calibration codes by using the software Matlab R2022b. In addition, we used the Matlab parallel computing toolbox to speed up the computation with the parpool (“Processes,” 20) option on the workstation.

For implementing the PBT (forecasting), we used two mobile laptops equipped with an Intel Core i7 processor with 8 and 12 cores, respectively, with 16 Gb of RAM without parallel computing.

## 3. Results

### 3.1. General modeling framework calibration results

#### 3.1.1. Data processing results

**Figure 3:**
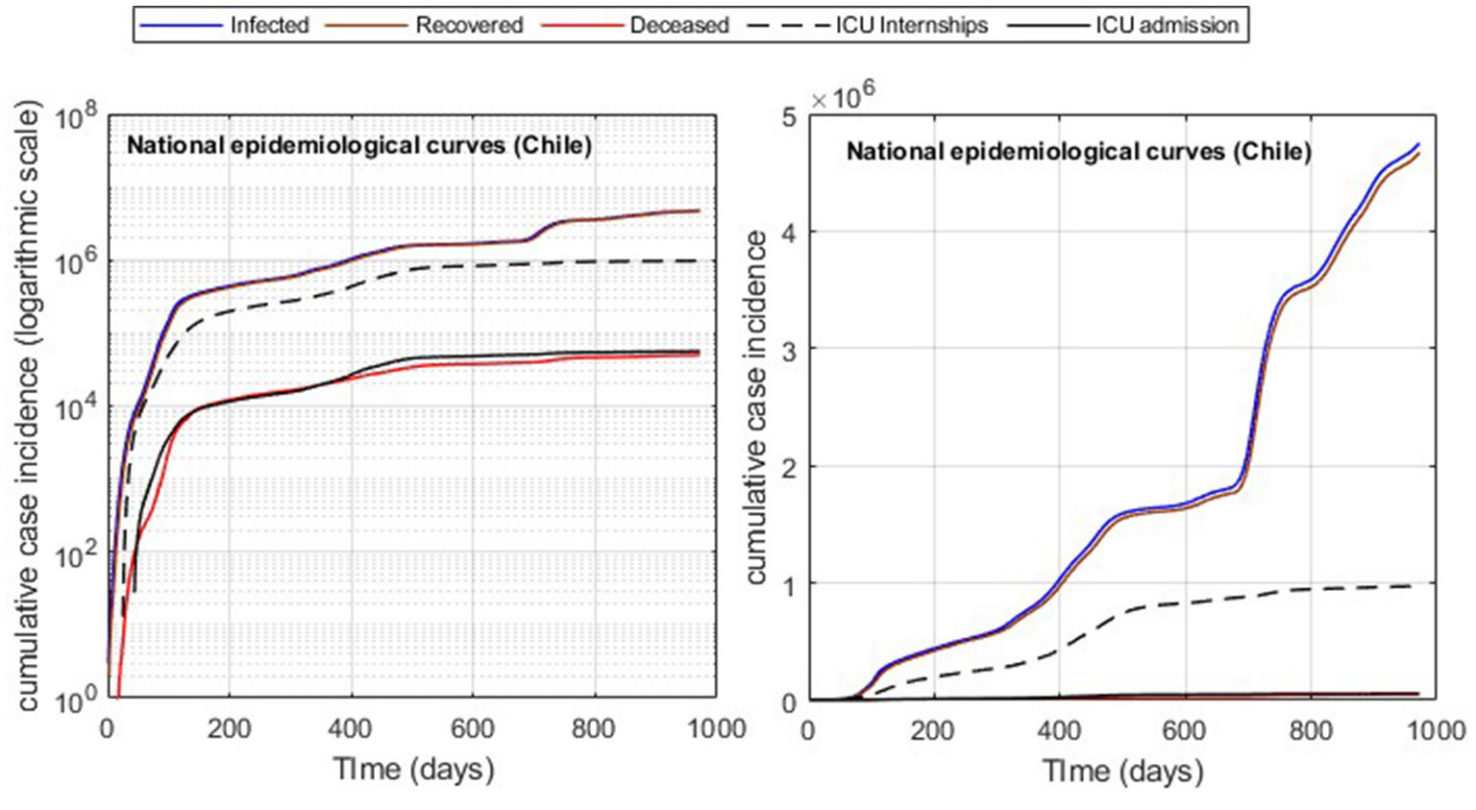
Cumulative incidence curves (Chile). Logarithmic and raw data, respectively.

Data processing shows that the highest correlated variables are *I* and *R*, followed by ICU admission with ICU hospitalized and deceased with ICU hospitalized (Figure 3). The two latter are the most relevant indicators of the impact of the pandemic on the health system, which justifies our choice of privileging the forecasting performance in *U*, as made in Equation (12).

In addition, the optimization results strongly depend on the parameters' initial values and the fitted data quality. By applying moving averages of 14 days, we reduced abrupt slope changes of the actual epidemiological curves (Figure 1), improving results with information loss of less than 2% of incidences for both infected and ICU. In addition, using moving windows combined with the cumulative distribution curves (Figure 3) reduces the number of steps of the Matlab dde23 solver (for non-linear DDE systems) because the slopes are always positive and not as steep as for daily curves. In this way, our method allows for calibrating parameters with accumulated and daily curves, where the cumulative data is helpful in efficiently fitting from the pandemic's beginning to any subsequent location and day to perform block tracking.

#### 3.1.2. Calibration results

The percentage decrease technique, described in Section 2.2.2.4, ensures the overall method stability and convergence by preventing the NaN appearance (NaN means “Not a Number”) when overflow occurs (computational numerical limit exceeded). NaNs are propagated in the model's history function, making the optimization solver lsqnonlin require more time to find new feasible points and often diverge. Using different ω values in $\theta^{\left(0\right)}:=\omega \cdot {\text{UB}}_{\theta }$, one may generate suitable initial curves for *I* and *U* below the epidemiological curves. For small ω values (ω ∈ [1*e* − 4, 1*e* − 2]), the initial curve will approximately be a straight line, obtained with government data, and greater than zero. By applying this simple but effective technique, our careful optimization algorithm implementation ensures stability and convergence for the dde23 solver and the lsqnonlin optimization subroutine within the range of suitable parameters, as described in Section 2.2.2.3.

Furthermore, to speed up the computation, we performed a one-step initial optimization defining the real positivity rate *f*(*t*) = 1. This was the same as taking *I*(*t*) = *I*_*r*_(*t*), i.e., all the actual infected are reported on the entire curve and storing the resulting ω as a checkpoint. Then, when required to optimize the curve over any time interval, the obtained ω was used as the initial value, starting the computation closer to the optimum.

**Figure 4:**
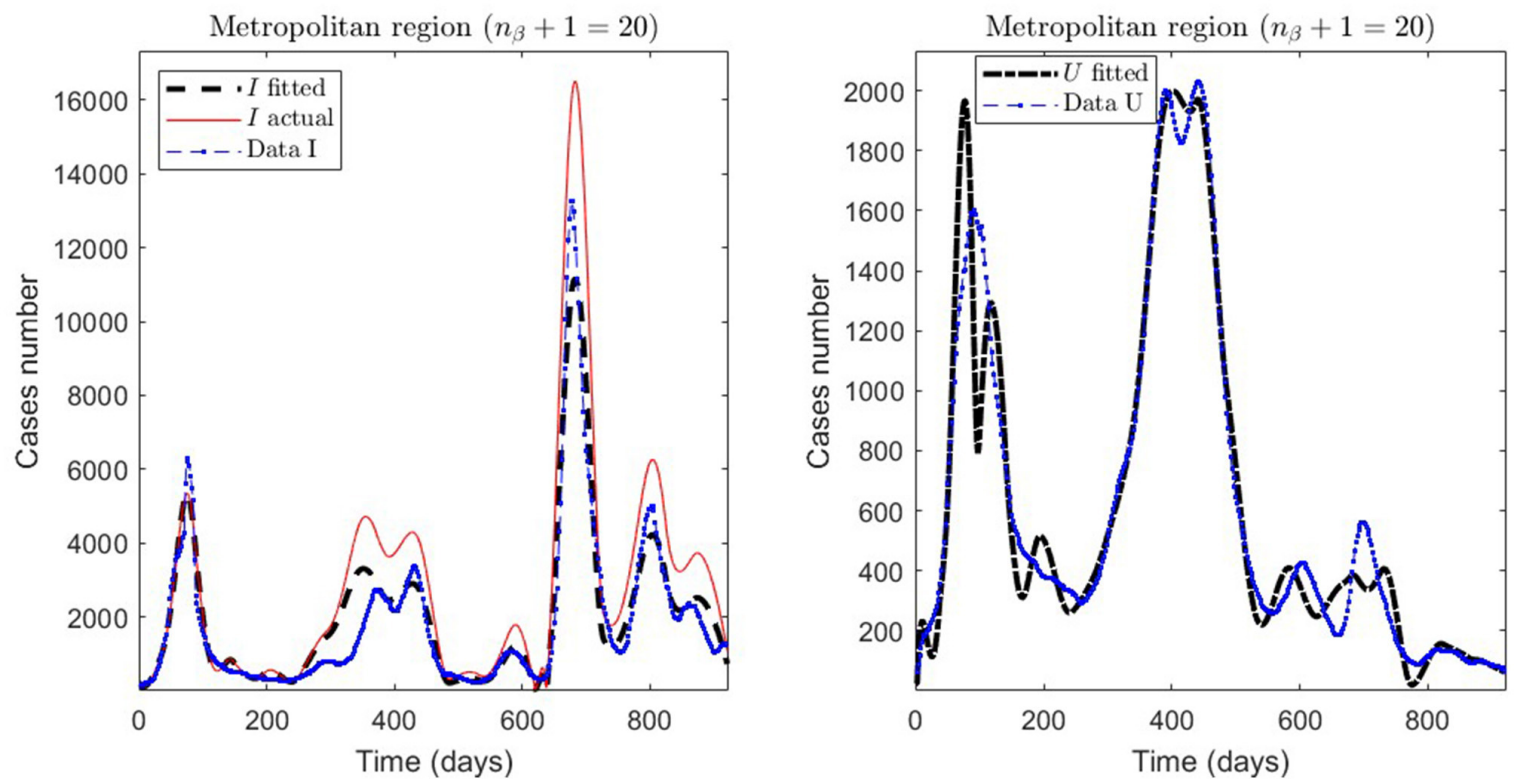
Fitted incidence curves in the Metropolitan region, Chile, 1 April 2020 to 8 October 2022.

**Figure 5:**
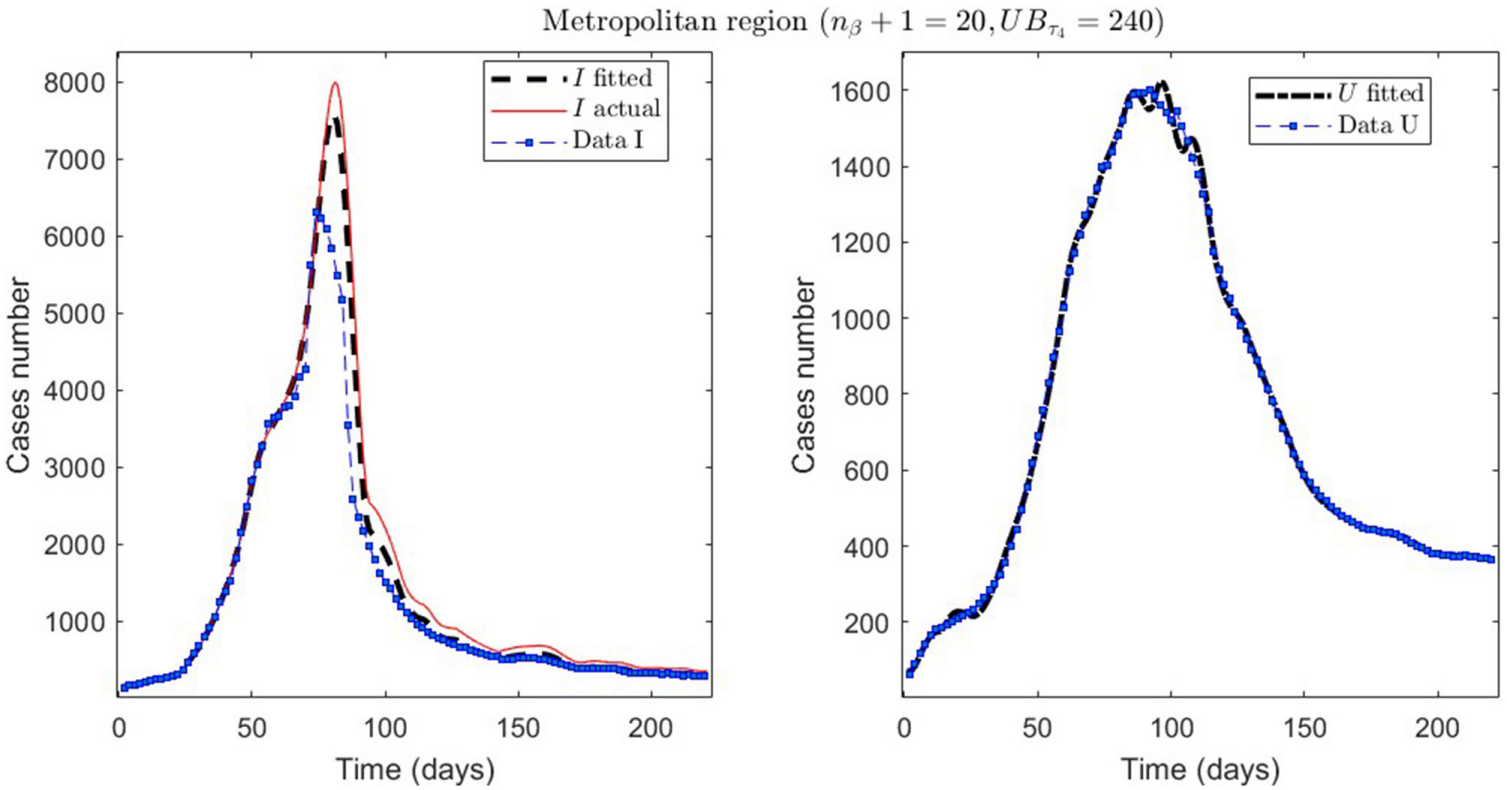
The first epidemiological wave in the Metropolitan region, Chile. 1 April 2020 to 7 November 2020; UB_τ_4__ = 240 (unrestricted immunity duration).

**Figure 6:**
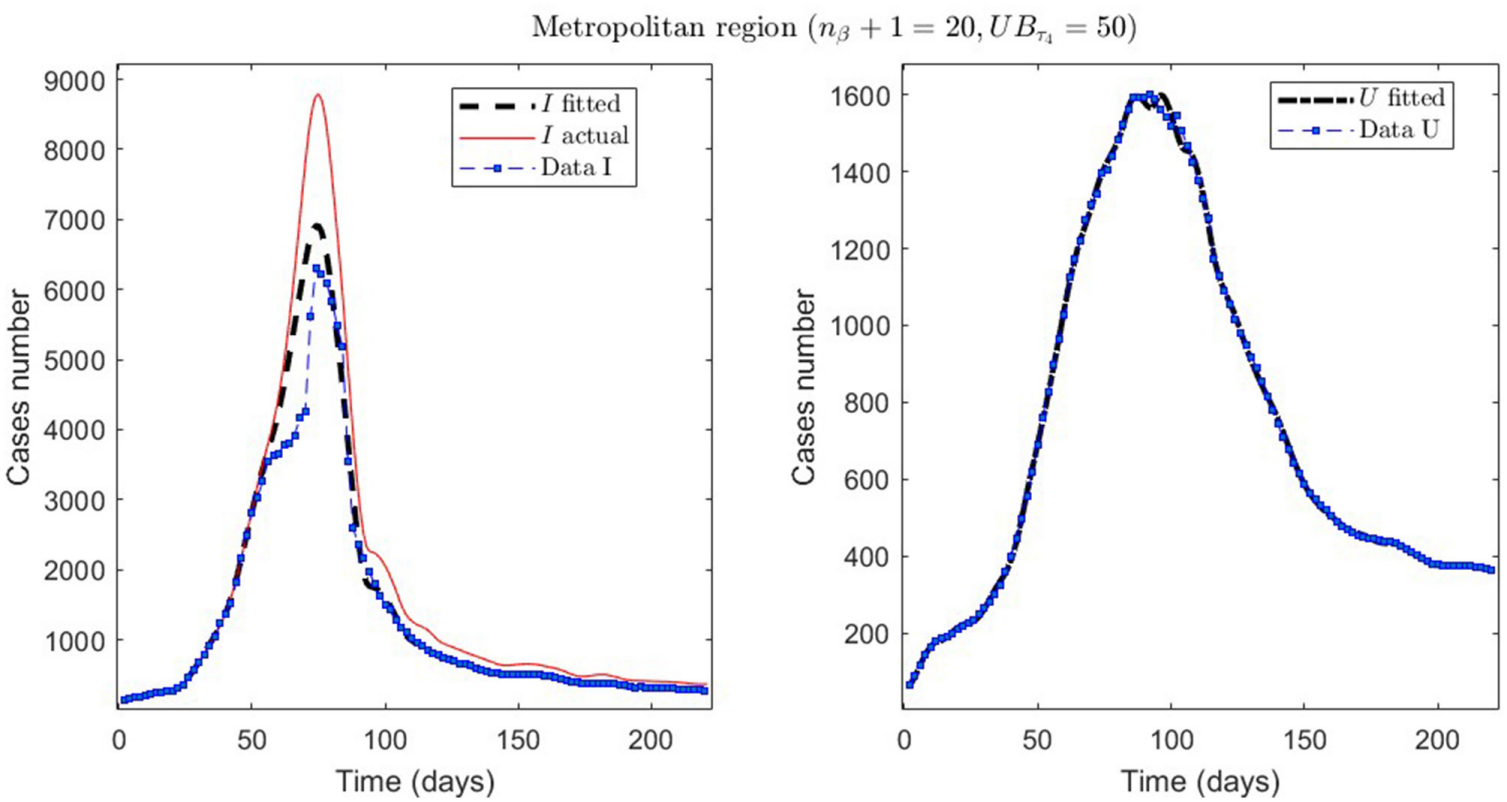
The first epidemiological wave in the Metropolitan region, Chile. 1 April 2020 to 7 November 2020; UB_τ_4__ = 50 (immunity duration restriction).

**Figure 7:**
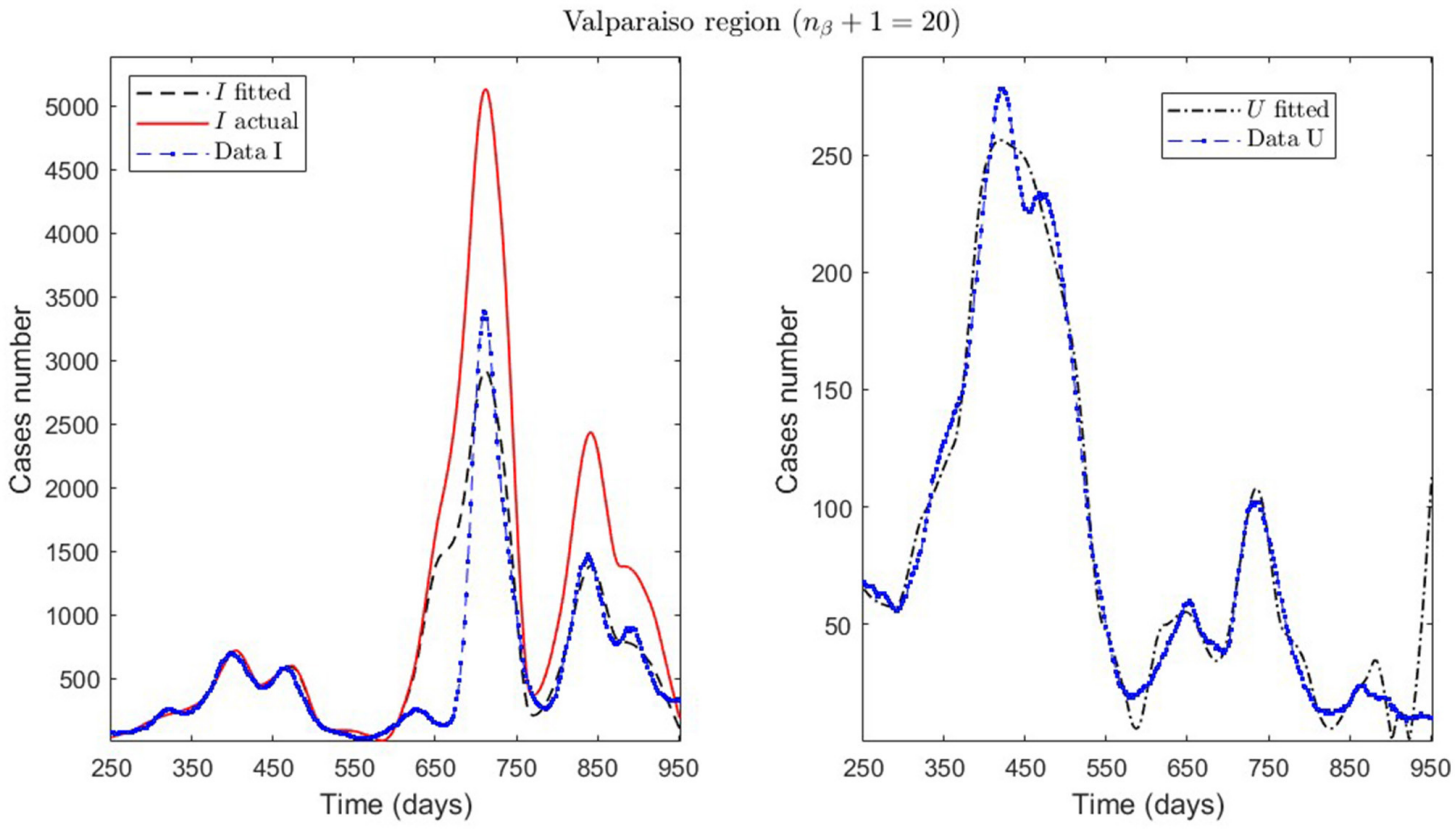
Fitted incidence curves in the Valparaíso region, Chile. 7 October 2020 to 8 October 2022; UB_τ_4__ = 240 (unrestricted immunity duration).

**Figure 8:**
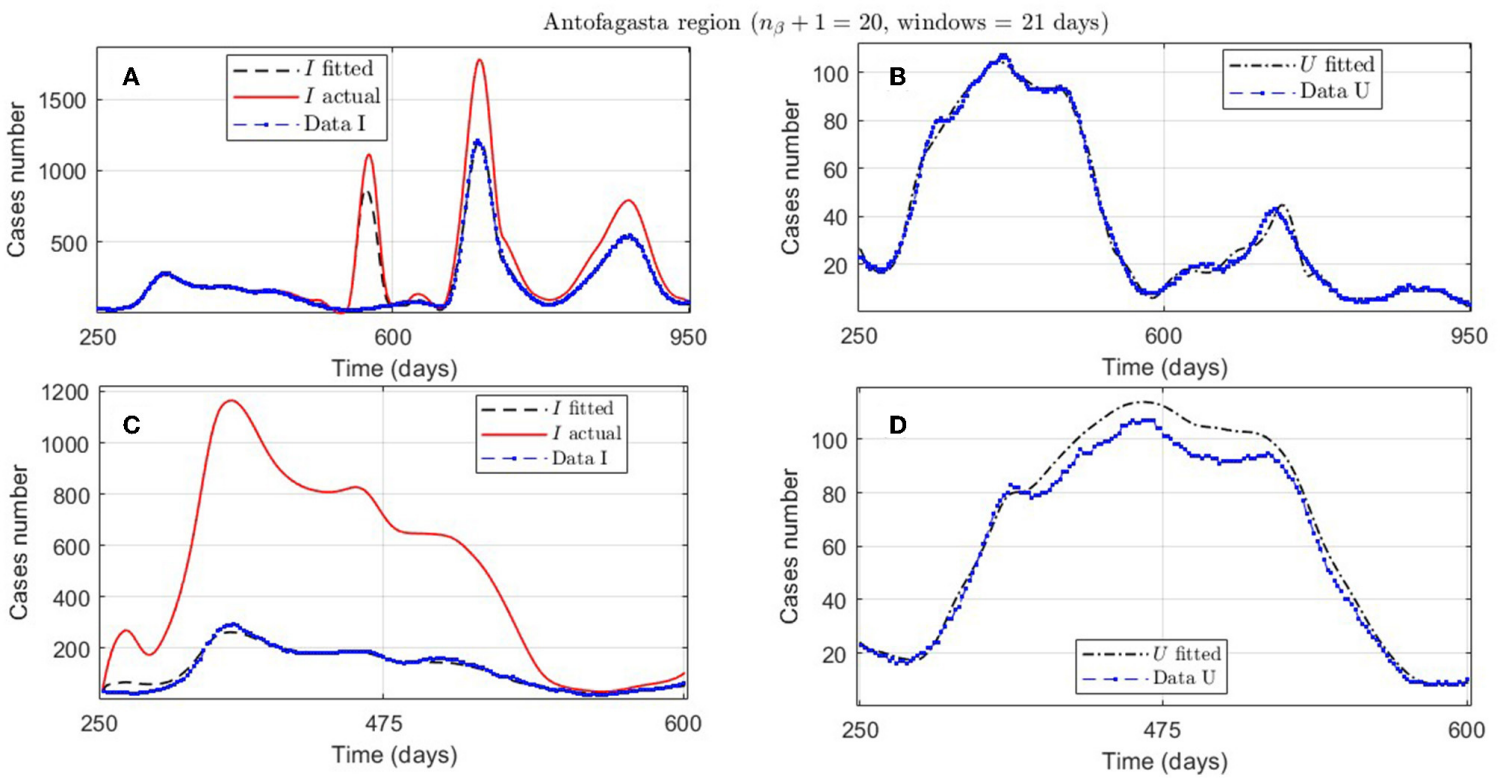
Fitted incidence curves in the Antofagasta region, Chile; UB_τ_4__ = 240 (unrestricted immunity duration). **(A, B)** 7 November 2020 to 8 October 2022; **(C, D)** 7 November 2020 to 23 October 2021.

**Figure 9:**
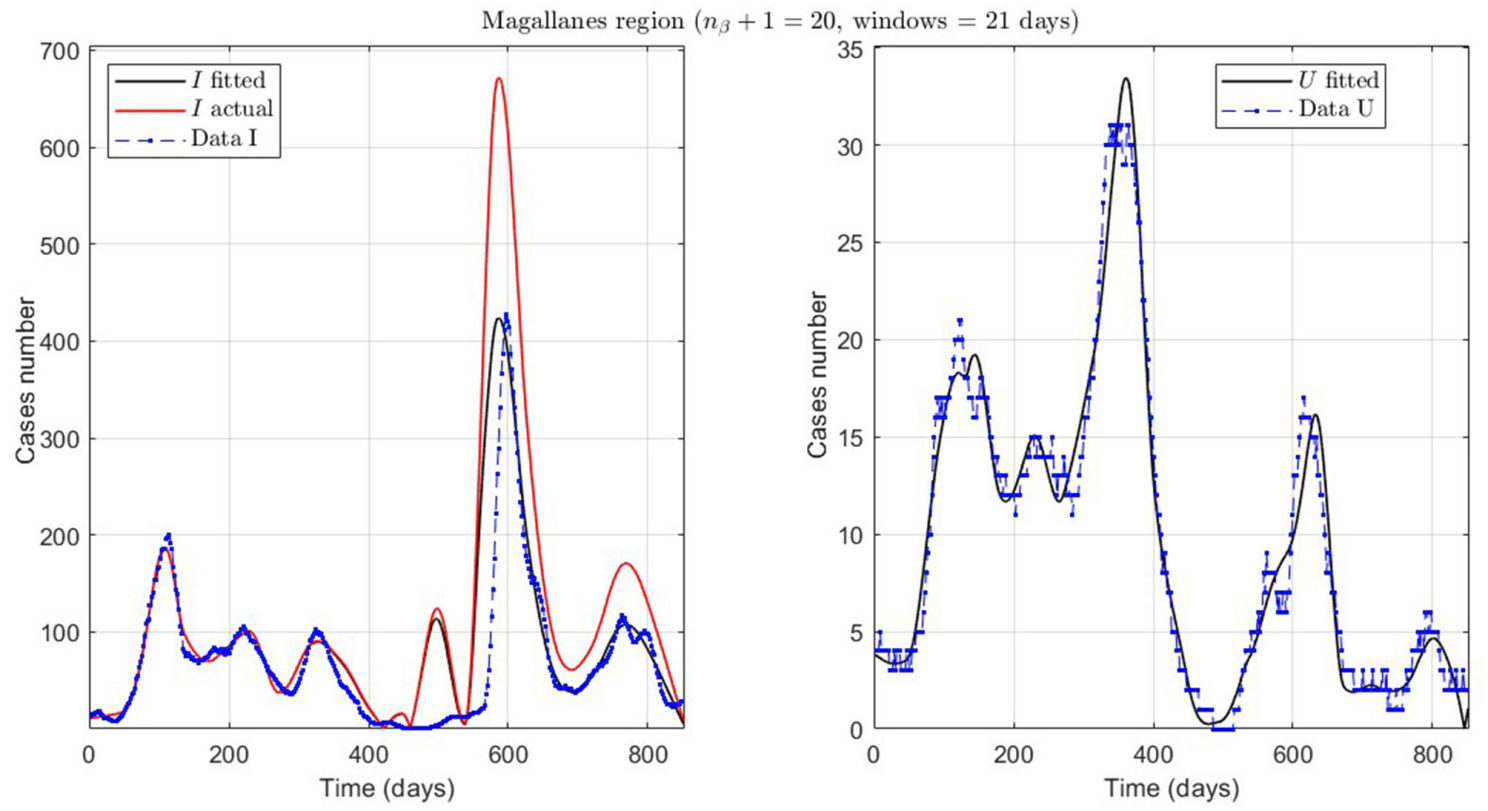
Fitted incidence curves in the Magallanes region, Chile. 10 June 2020 to 8 October 2022; UB_τ_4__ = 240 (unrestricted immunity duration).

In Table 2, we present the optimization results for several datasets located throughout all of Chile: the Metropolitan region, Valparaíso region (center), Antofagasta region (northern), and Magallanes region (southern), designated by the identifiers MR, VAL, ANT, and MAG in the Id column. We plotted the corresponding curves in Figures 4–9. In addition, Table 2 shows the results concerning a study on the immunity duration in different time intervals to assess vaccination. We obtained the calibration results by varying the upper bound UB_τ_4__ of the parameter τ_4_ for a dataset that contains the MR's first wave; see rows MR.2-MR.3 in Table 2 and Figures 5, 6. The interpretation was that the least mean relative error for *I* and *U*, $\bar{{\text{RE}}_{I}}$ and $\bar{{\text{RE}}_{U}}$, implied that the corresponding τ_4_ value was the most probable for the respective dataset. This τ_4_ variation is helpful for the analysis, simulation, and evaluation of epidemiological scenarios where, for example, a better fit for τ_4_ larger means a high vaccine immunity duration.

**Table 2:** Model results for different ω.

**Id**	**Fraction ω**	**Curve/window**	** $\bar{{\text{RE}}_{I}}$ **	** $\bar{{\text{RE}}_{R}}$ **	** $\bar{{\text{RE}}_{ICU}}$ **	***n*_β_ + 1**	***t* (days)**	**UB_τ_4__**
MR.1, Figure 4	1e-03	cum/2 weeks	3.2808*e* − 01	4.1021*e* − 01	1.8342*e* − 01	20	[30, 950]	240
MR.2, Figure 5	1e-03	cum/2 weeks	8.2663*e* − 02	1.4144*e* − 01	1.99823 − 02	20	[30, 250]	240
MR.3, Figure 6	1e-03	cum/2 weeks	4.5210*e* − 02	1.6722*e* − 01	9.4964*e* − 03	20	[30, 250]	50
VAL.1, Figure 7	1e-03	cum/3 weeks	1.0394*e* + 00	6.9195*e* − 01	7.9057*e* − 01	20	[250, 950]	240
ANT.1, Figures 8C, D	3.5e-03	daily/3 weeks	1.8747*e* − 01	1.0747*e* + 00	8.2074*e* − 02	20	[250, 600]	240
MAG.1, Figure 9	2.5e-04	cum/2 weeks	9.9075*e* − 01	3.2812*e* + 00	3.4139*e* + 00	20	[100, 950]	240

From Table 2, for example, by decreasing the upper bound of τ_4_, comparing the results between MR.2 and MR.3 (through $\bar{{\text{RE}}_{I}}$ and $\bar{{\text{RE}}_{U}}$), we observe that τ_4_ is small, which could be interpreted as correct, since in the MR's first wave there was no vaccination and therefore no immunity due to it.

General epidemiological modeling with cumulative data allows us to fit the complete epidemiological curve for analytical purposes (MR.1 in Table 2) with slightly less precision. In some cases, it was necessary to increase the moving window size to handle abrupt changes of derivative in the curves or optimize with other fractions of the upper bounds (different values of ω). Although the error obtained may increase, these approximations (Val.1 in Table 2) should be considered since they may indicate a data problem or a change in the epidemiological scenario. The fact that these cases of higher error occur only for the data from those infected is another reason to focus more on the number of ICU patients plus deceased.

An example of the cases explained above is the Antofagasta region, the results from which are quantified with the ANT.1 identifier in the Id column in Table 2 and depicted in Figure 8A. We observed a wave not reported in the data for *t* ∈ [250, 950]. In addition, Figure 8C depicts that the model fits data for *t* ∈ [250, 600], with low error for *I* and *U*, even if the actual infected (calculated by the model) was much higher than reported.

Finally, we present the case of the Magallanes region (MAG.1 in Table 2), a zone located in the extreme south of Chile with very few inhabitants and, therefore, little data. Despite this, the model also fits curve *U* reasonably, despite a more significant error, where the relevance is to interpret the curve's trend.

### 3.2. Parametric bootstrap technique results

In this section, we want to show the predictive capabilities of our general modeling framework. For that, we applied the PBT for the 280–530 time interval in the Metropolitan region (MR), which encompasses the MR's second wave, and the prediction period was 12 weeks.

First, we demonstrate the results obtained using the careful parameter optimization for fitting general epidemiological modeling to the actual data by minimizing the sum of squares (7) (step 1 of the algorithm described in Section 2.2.3). A code run with this method with 100 iterations took around 14 min in an Intel Core i7 processor with eight cores and 16 Gb of RAM.

**Figure 10:**
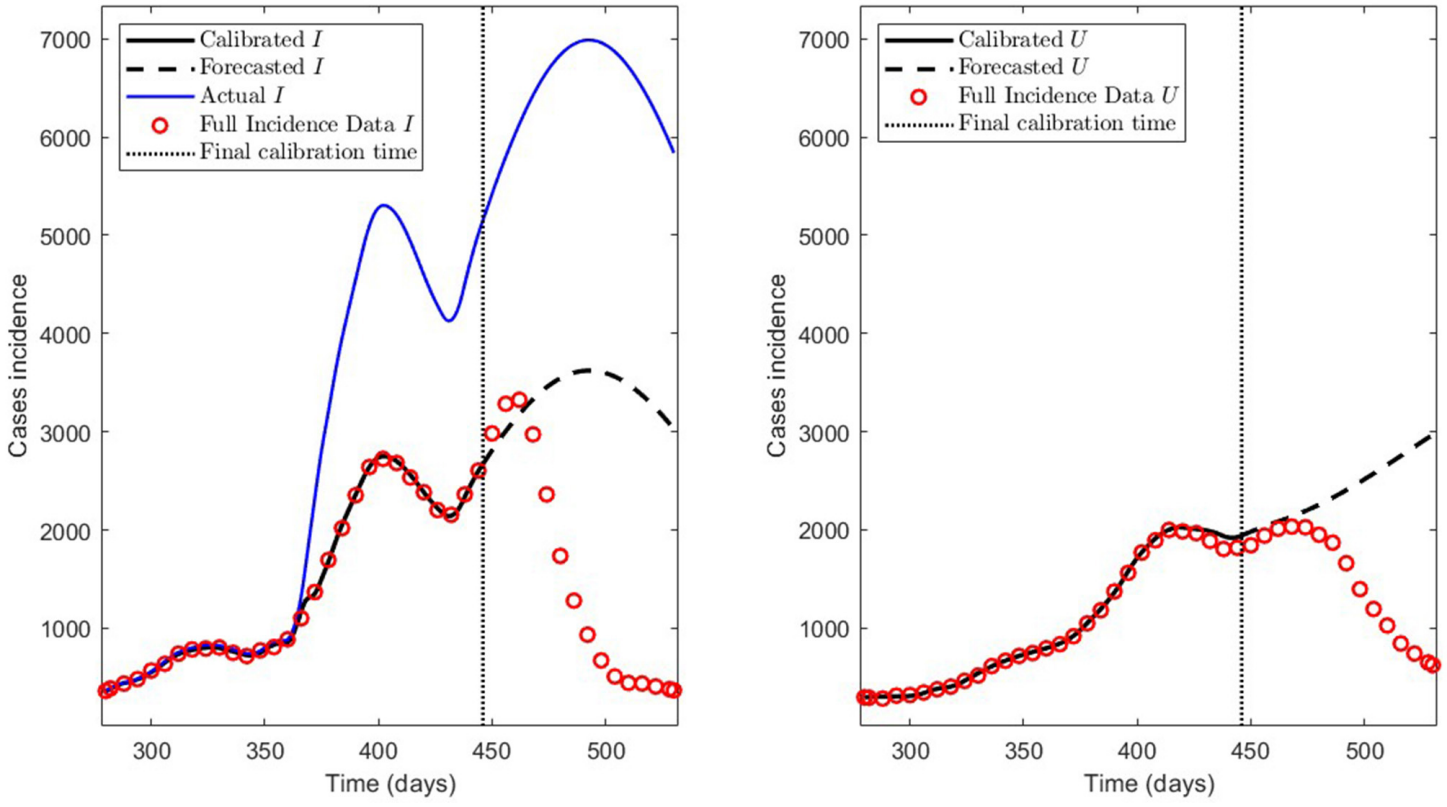
Incidence curves obtained by least-squares. 7 December 2020 to 14 August 2021, encompassing the Metropolitan region's second wave in Chile.

We summarize the numerical results in Table 3 and depict the least-squares fitted model curves $F\left(t_{j},\hat{\theta }\right)$ (Equation 9) in Figure 10. The corresponding error evaluated in the non-linear LSE $\hat{\theta }$, according to Equation (12), is $E\left(\hat{\theta }\right)=4.3387e-01$.

**Table 3:** Least-squares model results.

** 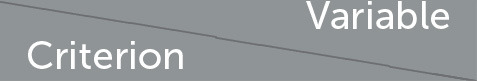 **	***I***	***U***
FP	6.3983e-01	4.3173e-01
RMSE	4.3539e-01	3.3227e-02

Second, we show the model results obtained through the PBT to achieve a better fitting and forecasting performance (the entire algorithm is described in Section 2.2.3). The PBT is computationally intensive since the code runs took around 24/16 h on one/two high-performance laptops, as described in Section 2.2.4.2. Using the two laptops, we calculated 50 parameter estimates to obtain the *M* = 100 bootstrap parameter realizations for fitting general epidemiological modeling to every synthetic dataset generated by a normal distribution with moderate variance relative to the least-squares curves $F\left(t_{j},\hat{\theta }\right)$ [Equation (9)], as explained in Section 2.2.3. Then, we constructed the best parameter estimate [${\hat{\theta }}_{\bar{k}}$ defined in (14)] and the PI [${\text{LB}}_{\hat{\theta }}$ and ${\text{UB}}_{\hat{\theta }}$ defined in (11)]. With these estimates, we plotted the respective curves for infected persons *I*_*r*_ and the sum of ICU patients and those who were reported dead *U*_*r*_.

**Figure 11:**
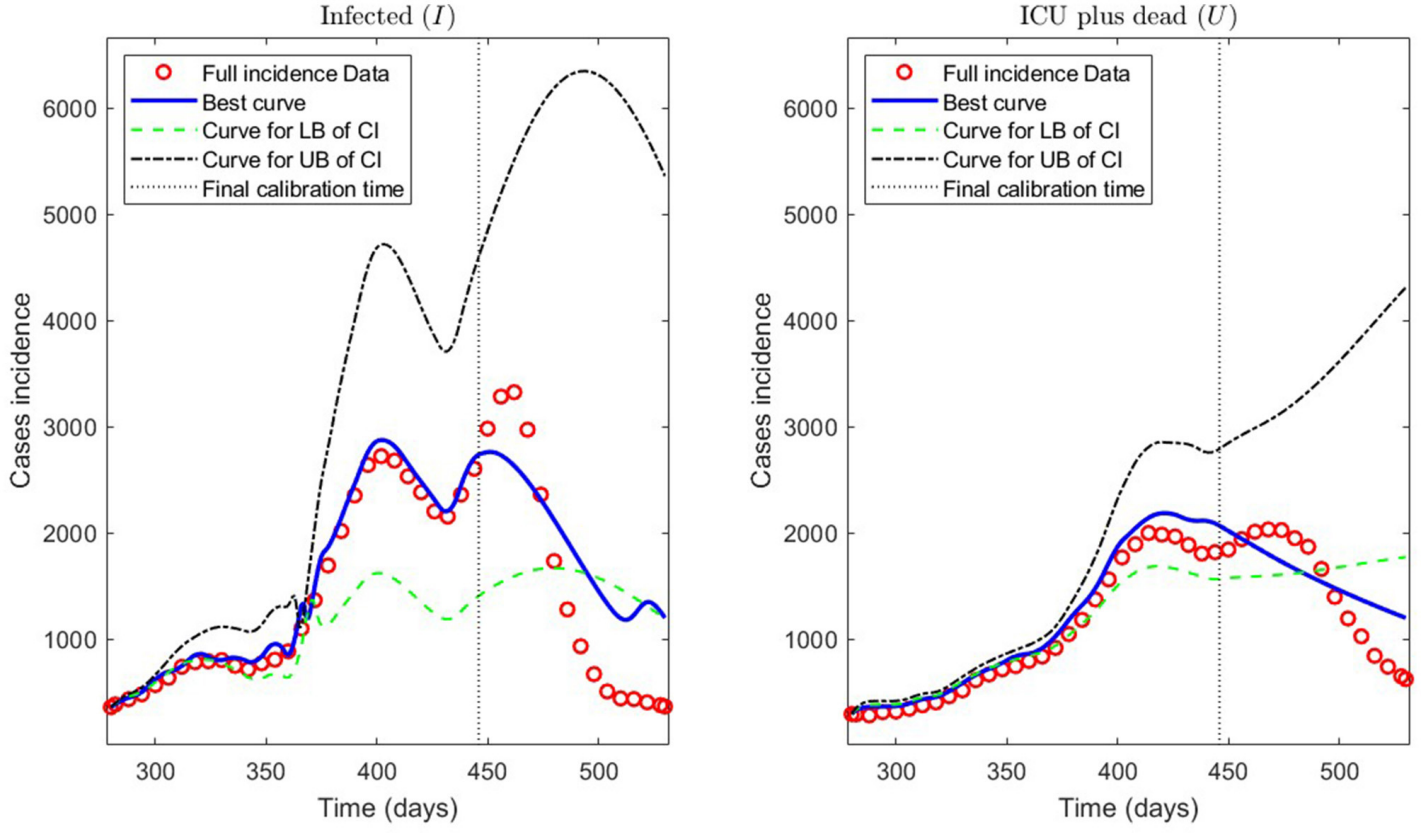
Incidence curves obtained by the PBT. 7 December 2020 to 14 August 2021, encompassing the Metropolitan region's second wave in Chile.

Figure 11 depicts the model results for synthetic datasets constructed as explained above. The minimum PBT error was $E\left({\hat{\theta }}_{\bar{k}}\right)=2.8775e-01$, calculated according to Equation (14). Finally, the parameter estimates uncertainty, corresponding to the curves plotted in Figure 11, ranged between 0.02 and 18.92% with a mean of 4.61% measured in normalized standard error (NSE) in percent, as defined in Equation (11b).

### 3.3. Assessing the impact of vaccination

We compared different values of the parameter τ_4_ in the range of 60–240 days to assess the impact of vaccination on immunity. For τ_4_ values spaced every 30 days, and ranging between 60 and 240 days, we obtained the results summarized in Table 4, which shows the minimum PBT error $E\left({\hat{\theta }}_{\bar{k}}\right)$ given in (14), the mean of the NSE ($\bar{\text{NSE}}$) given in (11b), and the weighted error WE given in (15).

**Table 4:** Model results for different values of τ_4_.

***τ***_**4**_	** $E\left({\hat{\theta }}_{\bar{k}}\right)$ **	** $\bar{NSE}$ **	**WE**
**60**	**2.7309e-01**	**4.9037e-02**	**1.9840e-01**
90	2.8199e-01	4.9866e-02	2.0462e-01
120	2.9898e-01	5.3788e-02	2.1725e-01
150	2.9882e-01	6.2228e-02	2.1995e-01
180	2.8646e-01	5.4880e-02	2.0926e-01
210	3.4228e-01	5.1622e-02	2.4539e-01
240	3.5367e-01	6.2833e-02	2.5672e-01

τ_4_ is the mean immunity duration by vaccination; see Table 1.

$E\left({\hat{\theta }}_{\bar{k}}\right)$ is the minimum error of the parametric bootstrap technique (PBT); see Equation (14).

$\bar{\text{NSE}}$ is the mean normalized standard error (NSE); see Equation (11b), and

WE is the weighted error; see Equation (15).

From Table 4, we observe that the best value for τ_4_ was τ_4_ = 60 days since it yielded the least weighted error WE.

## 4. Discussion

Concerning quantitative tracking of COVID-19 dynamics, we can calibrate any dataset with our general modeling framework. In effect, we show the framework's calibration capabilities through several examples for different regions of Chile; see Table 2 and the corresponding plotted curves in Figures 4–9. In addition, we corroborated that immunity duration was short (τ_4_ ≤ 50 days, as shown in row MR.3 in Table 2) during the Metropolitan region's first wave (when there was no vaccination yet). Consequently, our general modeling framework provides a flexible tool for studying the dynamics of any transmissible disease and assessing vaccination, despite the vaccination deficiencies and data limitations discussed in Sections 2.1.2.1, 2.2.4.1. To do so, it suffices to have time-series observations for the number of infected persons and ICU patients and to find a suitable range of initial parameters meaningful from the epidemiology viewpoint. Then, the percentage decrease technique allows us to find a unique range of parameters proper for every studied dataset, which provides overall method numerical stability and convergence, as shown in this article.

Concerning the framework's predictive capabilities, we applied the PBT to a dataset encompassing the Metropolitan region's second wave. The results shown in Table 3 and depicted in Figure 10, show that fitting the model to processed data by least-squares produces a forecasting performance for variables *I* and *U* that could be better (even more for *U*), despite their respective fitting performance (RMSE) being excellent. From the PBT implementation, we found that the parameter uncertainty range, evaluated through the percentage NSE, needed to have reliable parameter estimates (between 0.02 and 18.92% with a mean of 4.61%), so the number of bootstrap samples *M* = 100 was suitable. In addition, Figure 11 shows an outstanding forecasting performance for variables *I* and *U* (even more for *U*) with an error of 1.5 times less than for the usual least-squares method $\left(E\left(\hat{\theta }\right)\approx 1.5E\left({\hat{\theta }}_{\bar{k}}\right)\right)$. Therefore, our general epidemiological framework can accurately predict the ICU curve trend in the medium term (12 weeks). However, a limitation to achieving such a good prediction performance is that the PBT is costly from a computational viewpoint.

In Figure 11, we observe that the segmented curves (green lines) are more significant than the filled curves (blue lines) in specific time intervals, despite that the parameter vector of the firsts [evaluated at the lower bound of the PI; Equation (11a)] is smaller than the one of the seconds [evaluated at the best parameter vector; Equation (14)]. It may happen since, according to Equations (5b) and (5d), the curves for variables *I* and *U* will be significant if the γ_*IR*_(*t*), γ_*IU*_(*t*), and γ_*UR*_(*t*) rates are close to zero in some time intervals (which is the case of the segmented curves).

Concerning vaccination assessment relative to its immunity duration, from Table 4, we infer that τ_4_ > 60 values do not adapt to the dataset encompassing the MR's second wave. Therefore, the immunity duration should be less than 60 days. This exciting result implies a short immunity duration during the MR's second wave, which is not surprising since vaccination at that time was not yet widespread. In addition, this result would corroborate that the vaccination effect is not as significant as the immunity provided by natural infection, as discussed in Section 2.1.2.1. Indeed, the third wave magnitude (the most prolonged and steepest so far) shows that vaccination was relatively ineffective regarding protection against infection before it. However, one may think that vaccination manifests a positive effect by the time of the third wave, which we can infer from the fact that the ICU patient and death data are low compared to the infected data during that wave.

Our results rely on the piecewise linear reconstruction of time-varying parameters ***γ***_***IR***_, ***γ***_***SR***_, ***γ***_***RS***_, ***γ***_***IU***_, ***β***, and ***γ***_***UR***_. We could improve this arbitrary choice by assuming the mechanical laws of the transmission rate (***β***) or other rates as, for example, in (21).

## 5. Conclusions

This work attempts to implement a holistic and general modeling framework for quantitative tracking of the dynamics of any transmissible disease, focusing on accurately predicting the ICU curve trend in the medium term and assessing vaccination.

Implementing a careful optimization algorithm, we obtained outstanding results concerning quantitative tracking of COVID-19 dynamics for several processed datasets representing different regions of Chile and assessing vaccination. In addition, a parametric bootstrap technique allowed us to predict the ICU curve trend in the medium term accurately and assess vaccination. As a result, the scientific community could adapt our general modeling framework to evaluate the impact of combined vaccination and NPI strategies for COVID-19 or any transmissible disease in any country and help visualize the potential effects of implemented plans by policymakers.

In conclusion, the two main lessons are that we must be prepared to face another pandemic like COVID-19 and that it is more important to make more effort to follow up the ICU patients, which are highly correlated with dead confirmed by COVID-19.

To tackle the first lesson, in future work, we want to improve the computational cost of the parametric bootstrap technique or use another technique more efficiently. The aim would be to reconstruct epidemiological curves to predict the combined NPIs and vaccination policies' impact on the ICU curve trend in real time, providing scientific evidence to help anticipate policymakers' decisions.

## Data availability statement

Publicly available datasets were analyzed in this study. This data can be found here: https://github.com/MinCiencia/Datos-COVID19.

## Author contributions

PC contributed to conceptualization, formal analysis, investigation, methodology, project administration, software, supervision, validation, writing—original draft, review, and editing. OR-D contributed to conceptualization, formal analysis, investigation, methodology, software, validation, and writing—review and editing. CC contributed to theoretical analysis, supervision, and writing—review and editing. All authors contributed to the article and approved the submitted version.
